# Unfavorable cardiovascular risk profile without increased event prevalence in late-onset Pompe disease: an individually matched cohort study

**DOI:** 10.1186/s13023-026-04400-8

**Published:** 2026-05-27

**Authors:** Harmke A. van Kooten, Danny van Zoest, Michelle Michels, Nadine A. M. E. van der Beek, Esther Brusse, Ans T. van der Ploeg, Janneke G. Langendonk, Pieter A. van Doorn, Margreet A. E. M. Wagenmakers

**Affiliations:** 1 Department of Neurology, Center for Lysosomal and Metabolic Diseases, Erasmus MC, University Medical Center Rotterdam, Rotterdam, The Netherlands; 2 Department of Internal Medicine, Center for Lysosomal and Metabolic Diseases, Erasmus MC, University Medical Center Rotterdam, Rotterdam, The Netherlands; 3 Department of Cardiology, Cardiovascular Institute, Thoraxcenter, Erasmus MC, University Medical Center Rotterdam, Rotterdam, The Netherlands; 4 Department of Pediatrics, Center for Lysosomal and Metabolic Diseases, Erasmus MC – Sophia Children’s Hospital, University Medical Center Rotterdam, Rotterdam, The Netherlands

**Keywords:** Late-onset Pompe disease, Cardiovascular risk factors, Cardiovascular disease, Hypertension, Electrocardiogram

## Abstract

**Background:**

It is unclear whether cardiovascular risk factors are more common or if cardiovascular disease occurs more frequently in adults with late-onset Pompe disease (LOPD) than in the general population. We investigated the prevalence of cardiovascular risk factors and cardiovascular events in adults with LOPD compared to an individually matched control cohort.

**Methods:**

Adults with LOPD were individually matched to control subjects, with the same sex, smoking status, age (± 5 years) and BMI (± 5 kg/m^2^). Assessments included measurement of height, weight, waist-hip circumference, 30 min blood pressure measurement, electrocardiogram, and blood and urine laboratory analysis. Medical history of cardiovascular risk factors and events were collected via questionnaires and patient records.

**Results:**

Seventy-eight patients (median age 55.9 years, BMI 25.1 kg/m^2^) and 78 control subjects (median age 55.3 years, BMI 25.6 kg/m^2^) were included. Patients had a higher resting heart rate (76 vs. 66 BPM, *p* < 0.001), larger waist circumference (93 vs. 90 cm, *p* = 0.011) and waist-to-hip ratio (0.91 vs. 0.86, *p* = 0.005). A greater proportion of patients than controls had a history of hypertension (32% vs. 14%; *p* = 0.026). More patients than controls (19 vs. 9%) experienced a cardiovascular event in the past, but this difference was not statistically significant.

**Conclusion:**

Patients with LOPD showed a less favorable cardiovascular risk profile compared to an individually matched control cohort. The prevalence of cardiovascular events did not significantly differ between the cohorts, although our study is likely underpowered to detect subtle differences due to the low number of events.

## Introduction

Late onset Pompe disease (LOPD) patients may have a higher risk of cardiovascular complications due to several interrelated factors. Glycogen accumulation in smooth muscle in arterial walls and endothelial cells can lead to structural and functional abnormalities such as increased arterial stiffness, which contributes to increased systemic blood pressure (BP) and subsequent cardiovascular risk [1–4]. Additionally, the altered vascular integrity may increase the risk of aneurysms, dissections, and other vascular anomalies [5–8]. Furthermore, the structural and electrical remodeling of the heart in LOPD, especially due to glycogen storage in the conduction system, may predispose patients to arrhythmias, even in LOPD where left ventricular hypertrophy (LVH) is not part of the typical phenotype [9, 10].

Beyond the direct impact of glycogen accumulation on vascular health, LOPD indirectly affects cardiovascular risk through reduced muscle mass. In the general population, decrease in muscle mass impairs glucose metabolism and insulin sensitivity, potentially leading to insulin resistance and glucose intolerance [11]. It also often results in increased adipose tissue, particularly visceral fat, promoting chronic inflammation and metabolic dysfunction [12]. Reduced muscle mass is associated with decreased physical activity and functional capacity, leading to a more sedentary lifestyle [13]. Additionally, it lowers overall metabolic rate and energy expenditure, potentially contributing to obesity [14]. Muscle tissue also plays a role in maintaining healthy blood pressure and supporting cardiovascular function, further linking muscle loss to increased cardiovascular risk [15]. Moreover, as the disease progresses, patients experience progressive skeletal muscle atrophy and diminishing exercise capacity, which could further increase the incidence of adverse cardiovascular events.

Currently, there is a lack of studies that systematically evaluate whether patients with Pompe disease have an increased cardiovascular risk profile, and whether such risk is primarily due to the direct effects of Pompe disease on vascular health versus indirect factors like reduced muscle mass. To clarify these issues, we conducted a cohort study with an individually matched control group.

## Methods

### Study design and setting

We conducted a cross-sectional, single-center cohort study with an individually matched control group. This study is embedded in a prospective, nationwide, study (MEC-2007-103; “Effects and health economic aspects of enzyme therapy in children and adults with Pompe disease”). The data were collected between September 2018 and February 2021 at the Erasmus University Medical Center in Rotterdam, which serves as the single reference center for Pompe disease in the Netherlands.

### Study population

All patients diagnosed with LOPD, aged 18 or older, were potentially eligible for inclusion in the study. Diagnosis was confirmed by enzyme analysis in leukocytes and/or fibroblasts and by mutation analysis. Patients with a history of another disease known to be associated with an increased risk of cardiovascular disease/events (e.g. genetical disorders in lipid metabolism, auto-immune disease, obstructive sleep apnea syndrome) or (previous) use of medication which increases cardiovascular risk (e.g. corticosteroids, chemotherapy) were excluded. Patients were individually (one-on-one) matched to a control subject with comparable sex, age (± 5 years), BMI (± 5 kg/m^2^) and smoking status, which was categorized (current smoker, smoking in the past, never smoked or quit smoking for over 10 years). For the control cohort, we asked partners, friends, or family members of patients to participate, as they are expected to share similar lifestyle and environmental factors. If no related control subject was available, employees of the Erasmus MC University Medical Center were included as control subject. The same exclusion criteria applied to both control subjects and patients. The aim was to include all eligible patients and an equal number of control subjects.

The medical ethics committee at Erasmus University Medical Center approved this study and all participants provided written informed consent.

### Procedures

The following procedures were performed: (1) Physical measurements: weight (in kilograms), height (in centimeters), waist and hip circumference (in centimeters) were measured; (2) Blood pressure (BP) measurement: systolic blood pressure (SBP), diastolic blood pressure (DBP), mean arterial pressure (MAP) and heart rate (HR); (3) Electrocardiogram (ECG): a standard 12-lead ECG was performed; (4) Biochemical analysis: plasma glucose, HbA1c, creatinine, urea, and lipid profile (triglycerides, total cholesterol, HDL-cholesterol, non-HDL cholesterol and LDL-cholesterol) and presence of microalbuminuria in urine were measured.

Blood pressure measurements were conducted according to the European Society of Cardiology (ESC) guideline for office BP measurements [16]. We measured BP using the validated automated blood pressure device Datascope Accutorr Plus over a 30-minute period at five-minute intervals. Measurements were performed in a quiet room, with participants seated and their back and arm supported. In LOPD patients receiving ERT, blood pressure was not measured immediately after the infusion. To calculate the average SBP, DBP, MAP and HR, the first measurement of the 30 min measurement was disregarded. Elevated blood pressure was defined as SBP ≥ 140 mmHg and/or DBP ≥ 90 mmHg [16, 17]. Due to the screening nature of our study, repeat out-of-office BP measurements or ambulatory blood pressure monitoring (ABPM) were not performed to confirm the diagnosis of elevated BP or hypertension.

In cases where the ECG demonstrated abnormalities, the patient or control subject was referred to a cardiologist for further evaluation, which included echocardiographic assessment. Left ventricular hypertrophy was determined according to the Sokolow-Lyon criteria, a widely accepted method for evaluating ECG-based markers of ventricular wall thickening [18]. All ECGs were evaluated by a cardiologist specialized in metabolic diseases (MM).

Cholesterol levels were assessed in non-fasting blood samples; current guidelines consider non-fasting total cholesterol and HDL-cholesterol measurements acceptable for cardiovascular risk assessment, with fasting measurement recommended only in specific situations, such as markedly elevated triglycerides [19, 20]. Total cholesterol, HDL-cholesterol, LDL-cholesterol and triglycerides were directly measured at the ISO15189 accredited laboratory (Erasmus MC, Rotterdam, The Netherlands) on the C702, cobas^®^ 8000 system (Roche Diagnostics, Rotkreuz, Switzerland). Non-HDL cholesterol and cholesterol/HDL-cholesterol ratio were derived from these measures.

### Questionnaire

Patients and control subjects were asked to fill out a questionnaire containing relevant questions related to cardiovascular risk factors: (1) Medical history, with special regard to a history of cardiovascular disease/events and/or risk factors (i.e. hypertension, DM, hypercholesterolemia, peripheral artery disease, kidney disease); (2) Smoking; (3) Alcohol and/or drug use; (4) Diet (with particular attention to foods known to influence blood pressure, such as licorice (root) and salt); (5) Medication use; (6) Family history of cardiovascular disease. For all self-reported vascular diseases, events, or risk factors, data were verified by obtaining the relevant correspondence from the treating physician. Daily salt intake was estimated using the “Zoutmeter” (“Salt meter” in Dutch), an online tool by the Dutch Kidney foundation (www.nierstichting.nl/zoutmeter).

### Statistical analysis

SPSS for Windows (version 25; SPSS Inc, Chicago, IL) was used for all statistical analysis. Data were expressed as median (range) unless otherwise specified. Since the patient cohort and control cohort were individually matched for various characteristics, differences between the cohorts were assessed using paired tests: the Wilcoxon signed-rank test for continuous variables and McNemar’s test for categorical data, with significance set at *p* < 0.05. We did not apply a correction for multiple testing because the analyses were primarily exploratory and intended to identify potential patterns. If missing data occurred, little’s MCAR test was used to assess if data was missing at random. This was done only for variables with more than 5% missing data (data missing for more than 3 people). Data appeared to be missing completely at random, therefore only observed data was used in analysis [21]. Due to the low number of cardiovascular events in this study, logistic regression could not be performed.

## Results

### Study population

**Fig. 1:**
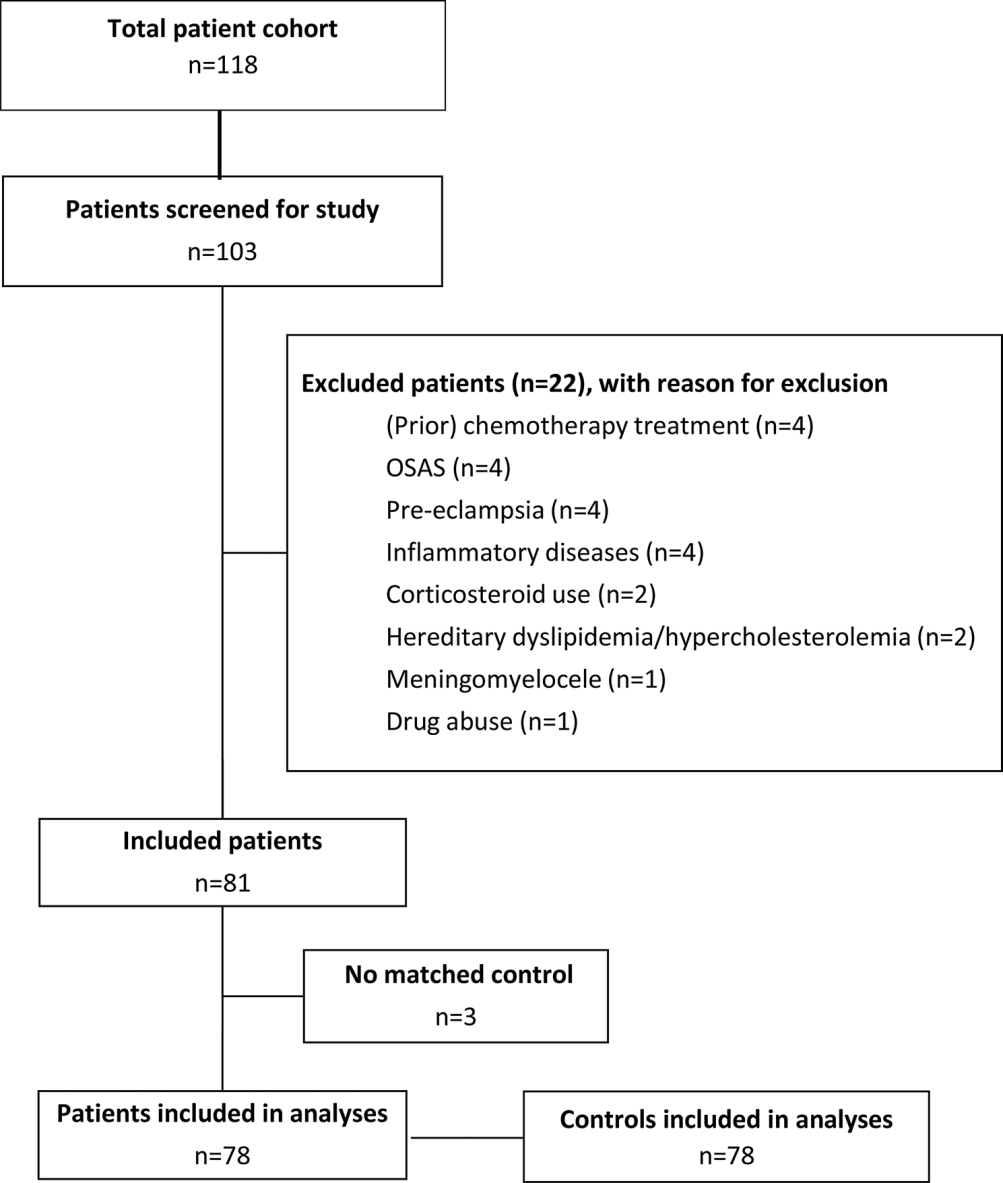
Flowchart of study population. OSAS = obstructive sleep apnea syndrome

Of the 118 patients in our cohort, 103 (87%) consented to participate and underwent screening for inclusion in the study. Among these, 22 were excluded due to conditions or treatments influencing cardiovascular risk (Fig. 1). For three patients, no suitable match could be found, resulting in 78 patient-control pairs available for analysis. Demographic data for the included patients and control subjects are presented in Table 1. Forty-four (57%) of the control subjects were employees of the Erasmus Medical Center. None of the matching criteria differed significantly between patients and control subjects (sex (43.6% vs. 43.6% males), age (55.9 vs. 55.3 years), BMI (25.1 vs. 25.6 kg/m2) and smoking status). The median duration of disease among patients (from symptom onset) was 18.7 years (range 0.4–56.4 years). Sixty-four patients (82%) were treated with enzyme replacement therapy (ERT), 61 patients received alglucosidase alfa (Myozyme) in a standard dose of 20 mg/kg every other week and three patients received avalglucosidase alfa (Nexviadyme) as part of a clinical trial. The median treatment duration was 9.25 years (range 0.1–14.5 years). Seventeen patients (22%) were wheelchair-dependent, and 25 patients (32%) used mechanical ventilation.

**Table 1:** Demographic and clinical characteristics

	Patients (*n* = 78)	Volunteers (*n* = 78)	*p*-value
Sex – male, *n* (%)	34 (43.6)	34 (43.6)	1.000
Age (years)	55.9 (24.7–82.8)	55.3 (21.6–80.0)	0.784
BMI (kg/m^2^)	25.1 (18.8–35.6)	25.6 (18.89-38.0)	0.109
*Smoking status*			
- Never or > 10 years ago, n (%)	66 (84.6)	67 (85.9)	1.000
- In the last 10 years, n (%)	9 (11.5)	8 (10.3)	1.000
- Current smoker, n (%)	3 (3.8)	3 (3.8)	1.000
Pack years	5.3 (0.0–50.0)	6.0 (0.0-62.5)	0.910
Disease duration (years)†	18.7 (0.4–56.4)	-	-
ERT – yes, n (%)	64 (82.1)	-	
Duration of ERT (years)‡	9.25 (0.1–14.5)	-	-
Wheelchair use, n (%)	17 (21.8)	0 (0)	-
Ventilator use, n (%)	25 (32.1)	0 (0)	-

Data are expressed as median (range), unless otherwise specified

† from onset of symptoms, ‡ *n* = 64

ERT= enzyme replacement therapy

### Outcomes of study procedures

Table 2 presents the study-derived measurements of outcomes influencing cardiovascular risk. Compared to control subjects, patients had a larger waist circumference (93 cm vs. 90 cm, *p* = 0.011) and a higher waist-to-hip ratio (0.91 vs. 0.86, *p* = 0.005), while hip circumference did not differ between the cohorts. During the 30-minutes BP measurement, no significant differences were found in SBP, DBP, or MAP between the cohorts. An elevated blood pressure (> 140/90 mmHg) was detected in 16 patients (21%), and in 12 control subjects (15%). Among the LOPD disease group, 59% (13/22) of patients on antihypertensive medication still had elevated BP during the 30-minutes measurement, compared to 29% (2/7) in the control cohort. Heart rate was higher in patients than in control subjects during datascope measurement (76 vs. 66 BPM, *p* < 0.001). Patients had significantly higher plasma glucose levels (5.5 vs. 5.4 mmol/L, *p* = 0.029) but this difference is not clinically relevant, HbA1c levels did not differ between the cohorts. Patients had lower plasma creatinine levels compared to control subjects (51 µmol/L vs. 77 µmol/L, *p* < 0.001) and consequently a significantly higher estimated glomerular filtration rate (eGFR), as calculated using the Chronic Kidney Disease Epidemiology Collaboration (CKD-EPI) method (109 mL/min vs. 84 mL/min, *p* < 0.001). Microalbumin levels in urine did not differ between the patient and control cohort (0.004 g/L vs. 0.004 g/L). On ECG, sinus bradycardia (26% vs. 5%, *p* < 0.001) and first-degree atrioventricular block (4% vs. 0, *p* = 0.016) were more prevalent among control subjects. There was no difference in the use of beta blockers between the cohorts. There were more LOPD patients with signs of LVH on ECG than control subjects (5% vs. 1%), but the difference did not reach statistical significance. No significant differences were observed between the cohorts in other findings.

**Table 2:** Outcomes of study procedures

	Patients *N* = 78	Controls *N* = 78	*p*-value
**Physical examination**			
Waist-to-hip ratio	0.91 (0.60–1.20)	0.86 (0.70–1.1)	**0.005**
Waist circumference (cm)	93 (54–122)	90 (70–114)	**0.011**
Hip circumference (cm)	103 (86–127)	104 (87–129)	0.289
**30 min blood pressure measurement**			
Systolic blood pressure (mmHg)	124 (99–191)	126 (102–195)	0.961
Diastolic blood pressure (mmHg)	76 (62–106)	76 (60–122)	0.674
Mean arterial pressure (mmHg)	95 (77–146)	94 (77–120)	0.757
Elevated blood pressure, n (%)	16 (21.9)	12 (15.4)	0.402
Heart rate (beats per minute)	75 (48–106)	66 (39–95)	**< 0.001**
**Laboratory testing**			
*Blood*			
Glucose (mmol/L)	5.5 (3.6–9.5)	5.4 (3.4–10.2)	**0.029**
HbA1c (mmol/mol)	36 (29–55)	37 (28–48)	0.272
Urea (mmol/L)	4.6 (2.7-9.0)	4.9 (2.6–8.6)	0.318
Creatinine (µmol/L)	51.0 (20.0–82.0)	77 (51–109)	**< 0.001**
eGFR (CKD-EPI, mL/min)	108.7 (82.6-151.8)	84 (55–124)	**< 0.001**
Triglycerides (mmol/L)	1.34 (0.54–4.77)	1.19 (0.41–5.47)	0.537
Total cholesterol (mmol/L)	5.0 (2.90–8.10)	5.10 (3.2–8.8)	0.627
HDL-cholesterol (mmol/L)	1.58 (0.90–3.14)	1.53 (0.83–2.57)	0.780
Non-HDL cholesterol (mmol/L)	3.39 (1.51–6.64)	3.47 (1.45–6.23)	0.707
LDL-cholesterol (mmol/L)	3.27 (1.33–6.56)	3.19 (1.39–6.05)	0.954
Cholesterol/HDL-cholesterol ratio	3.0 (1.9–6.8)	3.2 (1.8–6.8)	0.732
*Urine*			
Micro albumin (g/L)	0.004 (0.002-0.104)	0.004 (0.002-0.101)	0.640
ECG findings*			
Normal ECG, n (%)	58 (75)	48 (62)	0.063
Sinus bradycardia, n (%)	4 (5)	20 (26)	**< 0.001**
Left ventricular hypertrophy, n (%)	4 (5)	1 (1)	0.375
Right bundle branch block, n (%)	3 (4)	1 (1)	1.00
Incomplete right bundle branch block, n (%)	2 (3)	2 (3)	1.00
Intraventricular conduction delay, n (%)	3 (4)	2 (3)	1.00
Left anterior fascicular block, n (%)	2 (3)	1 (1)	1.00
Left bundle branch block, n (%)	1 (1)	-	1.00
First-degree atrioventricular block, n (%)	-	3 (4)	**0.016**

* ECG was missing for one patient

### Self-reported history of cardiovascular disease and risk factors

Self-reported cardiovascular disease/events and risk factors are summarized in Table 3. More patients than control subjects (19% vs. 9%) experienced a cardiovascular event in the past, but this difference did not reach statistical significance. Among these, three patients had a dilatation of the ascending aorta, whereas this was observed in only one control subject. Additionally, one patient had suffered a subarachnoid hemorrhage (SAH) due to a rupture of an aneurysm in the posterior inferior cerebellar artery. Twenty-five patients (32%) had a diagnosis of hypertension in their medical history, compared to eleven control subjects (14%) (*p* = 0.026). A higher percentage of patients was currently using antihypertensive medication compared to control subjects (28% vs. 9%, *p* = 0.004). The use of more than one antihypertensive drug was more frequent among patients than control subjects (10% vs. 5%). Three patients with LOPD (4%) had a previous diagnosis of DM, whereas none of the control subjects had a prior diagnosis of DM.

**Table 3:** Self reported history of cardiovascular disease and risk factors from questionnaire

	Patients *N* = 78	Controls *N* = 78	*p*-value
**Cardiovascular disease/events**			
Any cardiovascular disease/event, n (%)	15 (19.2)	7 (9.0)	0.115
Myocardial infarction, n (%)	0 (0)	1 (1.3)	-
Arrhythmias, n (%)	7 (9.0)	3 (3.8)	0.503
Bicuspid aortic valve, n (%)	0 (0)	1 (1.3)	-
Dilated ascending aorta, n (%)	3 (3.8)	1 (1.3)	0.625
Dilated aortic sinus, n (%)	1 (1.3)	1 (1.3)	1.000
TIA, n (%)	1 (1.3)	1 (1.3)	1.000
SAH from ruptured aneurysm, n (%)	1 (1.3)	0 (0)	-
Intermittent claudication, n (%)	2 (2.6)	0 (0)	-
**Cardiovascular risk factors**			
History of hypertension, n (%)	25 (32.1)	11 (14.1)	**0.026**
History of diabetes mellitus, n (%)	3 (3.8)	0 (0)	-
History of kidney disease, n (%)	1 (1.3)	0 (0)	-
History of hypercholesterolemia, n (%)	8 (10.3)	4 (5.1)	0.388
Family history of CVD, n (%)	28 (35.9)	29 (37.2)	1.000
**Medication**			
Antihypertensive medication, n (%)	22 (28.2)	7 (9.0)	**0.004**
Beta blocker use, n (%)	4 (5.1)	4 (5.1)	1.000
Antidiabetic medication, n (%)	3 (3.8)	0 (0)	-
Statin use, n (%)	4 (5.1)	2 (2.6)	0.625
**Diet**			
Alcohol use, n (%)	52 (66.7)	57 (73.1)	0.728
Amount of alcohol (glasses per week)	2 (0–25)	4 (0–20)	**0.026**
Licorice use, n (%)	43 (55.1)	49 (62.8)	0.377
Salt intake (g/day)	6.0 (2.0–15.0)	5.5 (2.0–15.0)	0.118

Data are expressed as median (range), unless otherwise specified

## Discussion

This large-scale single-center study is the first to compare the prevalence of cardiovascular risk factors and the occurrence of cardiovascular events in adults with LOPD to a individually matched control cohort. We found a higher resting heart rate and increased waist-to-hip ratio in LOPD patients. A larger proportion of patients than controls had a history of hypertension. More patients than controls (19% vs. 9%) experienced a cardiovascular event in the past, but this difference was not statistically significant. Our study is likely underpowered to detect more subtle differences due to the low number of events.

Pompe disease patients exhibited a significantly higher resting HR compared to control subjects. There are several factors that may attribute to this observed difference, such as reduced physical activity level, deconditioning and a higher fat mass percentage in patients with LOPD. An elevated resting HR is an established independent risk factor for cardiovascular disease and may thus contribute to an unfavorable cardiovascular risk profile [22, 23].

On ECG, sinus bradycardia was more prevalent in the control cohort, possibly due to better athletic conditioning. Other ECG findings previously reported in patients with LOPD, such as a shortened PR interval, RBBB, and indications of LVH, were not more prevalent in our patient cohort [24, 25]. However, due to the sample size, definitive conclusions cannot be drawn from these observations. Despite suggestions of higher Wolf-Parkinson-White (WPW) syndrome prevalence in LOPD patients, no patient in our study exhibited WPW syndrome on ECG examination [26].

Patients with LOPD had a significantly larger waist circumferences and higher waist-to-hip ratios compared to control subjects, potentially indicating increased visceral adipose tissue, which is associated with elevated cardiovascular disease risk [27, 28]. However, abdominal muscle weakness in patients with LOPD may lead to an increased waist circumference (and higher waist-to-hip ratio), thereby limiting its validity as a measure of visceral adiposity [29–31]. To accurately assess adipose tissue distribution LOPD patients, alternative imaging methods such as DEXA or MRI scans should be explored.

We found higher non-fasting glucose levels in LOPD patients compared to control subjects, but this was not a clinically relevant difference and within normal range. HbA1c levels were similar between groups, indicating comparable long-term glucose control. However, due to reduced muscle mass and impaired mobility, LOPD patients may have an increased risk of developing diabetes mellitus as they age, warranting regular screening for diabetes [32, 33].

Patients with LOPD showed lower serum creatinine levels, as a consequence of their reduced muscle mass. Since eGFR is calculated from creatinine levels, this is an unreliable measure for kidney function in these patients [34, 35]. A more reliable measure for renal function in patients with low muscle mass could be serum cystatin C, which is secreted by all nucleated cells, independent of muscle mass [36]. At the time of this study, cystatin C measurement was not part of routine clinical practice at our institution. However, it has now been incorporated into standard clinical follow-up for LOPD patients.

In our study, the self-reported prevalence of cardiovascular disease and related events did not significantly differ between LOPD patients and the matched control group. However, it is important to note that this finding may be influenced by the relatively small sample size and low event rate in both groups, which limits the statistical power to detect more subtle differences between the groups. The relatively young median age of our cohort (approximately 56 years) contributed to the low overall event rate, as advancing age is a well-established independent risk factor for cardiovascular disease [37–39]. We hypothesize that in our patients, the combination of Pompe disease and aging may elevate the risk of cardiovascular events beyond that observed in the general population over time. To further investigate this, long-term prospective follow-up studies are needed. On the other hand, patients with Pompe disease, especially those receiving ERT, typically undergo regular monitoring, including blood pressure measurements and laboratory assessments, which may facilitate early detection and management of cardiovascular risk factors, potentially mitigating the overall cardiovascular risk in this population.

We found that significantly more patients than control subjects had a prior diagnosis of hypertension, confirming earlier findings in our cohort [1]. Factors contributing to this increased hypertension prevalence may include reduced physical activity due to muscle weakness. Lack of physical activity can lead to hypertension through various mechanisms, including weight gain, increased sympathetic nervous system activity, and impaired endothelial function [40–42]. Furthermore, patients with LOPD have a higher fat mass, which may also contribute to the development of hypertension [43, 44]. Additionally, glycogen accumulation in arterial walls may exacerbate hypertension by increasing vascular stiffness [1]. We also found that more LOPD patients had an elevated blood pressure despite the use of antihypertensive medication and that more patients than control subjects needed more than one antihypertensive drug. However, conclusions should be interpreted with caution, as blood pressure was assessed by a single-visit office measurement only, whereas ESC guidelines recommend confirmation with out-of-office measurements (home or ambulatory blood pressure monitoring) or at least one repeat office measurement at a subsequent visit to establish (persistent) hypertension [16]. To mitigate cardiovascular risk, we suggest regular BP monitoring, including 30-minute measurements if abnormalities are detected, and early initiation of treatment. Lifestyle modifications such as dietary changes, alcohol moderation, weight management, and smoking cessation should be promoted [45]. Additionally, physical activity and (personalized) exercise training should be encouraged for LOPD patients, as they are found to be safe and beneficial [46–48].

We emphasize that for this study, we chose a set of screening instruments and tests that can be easily applied in daily clinical practice. By utilizing tests that require minimal time, resources, or specialized expertise, we aimed to increase the feasibility of early detection of risk factors. This approach enables the rapid identification of potential health issues, after which targeted additional investigations can be initiated if necessary. In this way, the selected tests contribute to efficient and accessible screening across various healthcare settings.

Cardiovascular risks in neuromuscular disorders (NMDs) vary by disease pathology, primarily manifesting in two major categories: cardiomyopathy (e.g. Duchenne/Becker muscular dystrophy, limb-girdle muscular dystrophies) and conduction defects with arrhythmias (e.g. Emery-Dreifuss muscular dystrophy, myotonic dystrophy) [49, 50]. In classic infantile Pompe disease cardiomyopathy is a hallmark of the disease [51]. In contrast, in LOPD, cardiovascular risk seems to arise more from systemic vascular pathology and metabolic factors than intrinsic cardiac muscle pathology. Similarly, in other NMDs, metabolic factors including obesity, insulin resistance, and chronic inflammation driven by reduced muscle mass, compound cardiovascular risks in addition to direct cardiac pathology [52, 53].

The main strength of our study is the one-on-one matching procedure, which considered age, sex, BMI, and smoking status, which are key contributors to cardiovascular disease risk [54–57]. One-on-one matching in cohort studies offers distinct advantages over group-level matching. By pairing each exposed participant with an unexposed participant who shares key characteristics, such as age and sex, one-on-one matching provides more precise control over confounding variables and enhances the comparability between groups. This approach reduces the influence of confounders on study outcomes and allows for more accurate and intuitive interpretation of results, while group level matching may leave residual confounding at the individual level. Furthermore, we excluded individuals with a history of diseases or medications that elevate cardiovascular risk from both patient and control groups to minimize confounding effects. A wide range of cardiovascular risk factors was investigated for a comprehensive assessment and all self-reported outcomes were carefully assessed by reviewing the medical records of both patients and control subjects.

There are several limitations to our study. First, over half our control cohort were Erasmus Medical Center employees, due to COVID-19 restrictions limiting patient access. Using healthcare workers as a control cohort may have influenced our findings, as their cardiovascular risk profile may not be representative of the general population. In previous studies, the results regarding cardiovascular risk in healthcare workers are conflicting, with some studies suggesting a “healthy worker effect” and others showing substantial burden of cardiovascular risk factors, likely related to occupational stress, shift work, and lifestyle factors [58–61]. As a result, comparisons with this control group may have attenuated or exaggerated differences in cardiovascular outcomes, depending on the direction of these biases. However, our matching methodology partially mitigates these differences. The other control subjects were relatives or friends of patients, as they are likely comparable with respect to shared environmental and lifestyle factors such as diet, household environment, and socioeconomic context [62]. However, other cardiovascular risk determinants may differ between patients and their relatives, particularly physical activity in those with a neuromuscular disorder, which we did not quantify despite its strong link to cardiovascular disease [63]. Nonetheless, patients in our center are encouraged to remain physically active, as this improves endurance, muscle strength, and muscle function [47]. Future studies should objectively measure physical activity, for example using actigraphy or including a control group with similar mobility limitations. Furthermore, although our questionnaires included several dietary components influencing cardiovascular risk, such as alcohol, salt, and licorice intake, a more comprehensive and structured dietary assessment would have strengthened the robustness of our analyses and conclusions. Also, we used BMI as a matching criterion, a common proxy for fat mass in population studies. However, in LOPD patients with reduced muscle mass, BMI may underestimate fat mass. Consequently, patients may have a higher fat mass than controls with the same BMI, which may in turn lead to underestimation of cardiovascular risk [43, 64]. Waist circumference, another easily measured proxy for fat mass, is also unreliable in Pompe disease, as noted previously. While DEXA scanning provides the most accurate assessment of fat mass, it is practically impossible to use as a matching criterion because numerous scans would be required to identify ideal matches. Bioelectrical impedance analysis (BIA) represents an alternative method for estimating fat mass, but it likewise requires screening a large number of potential controls, limiting its feasibility.

## Conclusions

We observed a higher resting heart rate and an increased waist-to-hip ratio in patients with LOPD compared to an individually matched control cohort. Also, more patients than controls had a history of hypertension. A greater proportion of patients than controls had experienced a cardiovascular event (19% vs. 9%); however, this difference did not reach statistical significance. The study was likely underpowered to detect subtle differences due to the low number of events. Nonetheless, as our original hypothesis anticipated a marked difference between the groups, the absence of such a difference may be interpreted as clinically reassuring for patients with LOPD.

## Data Availability

The data that support the findings of this study are available from the corresponding author upon reasonable request.
